# Using Cherenkov imaging to monitor the match line between photon and electron radiation therapy fields on biological tissue phantoms

**DOI:** 10.1117/1.JBO.25.12.125001

**Published:** 2020-12-09

**Authors:** Yi Li, Hongjun Liu, Nan Huang, Zhaolu Wang, Chunmin Zhang

**Affiliations:** a Chinese Academy of Sciences, Xi’an Institute of Optics and Precision Mechanics, State Key Laboratory of Transient Optics and Photonics, Xi’an, China; b Xi’an Jiaotong University, School of Physics, Xi’an, China; c University of Chinese Academy of Sciences, Beijing, China; d Shanxi University, Collaborative Innovation Center of Extreme Optics, Taiyuan, China

**Keywords:** Cherenkov luminescence imaging, radiotherapy, field matching, biological tissue, charge-coupled device

## Abstract

**Significance:** Due to patients’ respiratory movement or involuntary body movements during breast cancer radiotherapy, the mismatched adjacent fields in surface exposure regions could result in insufficient dosage or overdose in these regions, which would lead to tissue injury, excessive skin burns, and potential death. Cherenkov luminescence imaging (CLI) could be used to effectively detect the matching information of adjacent radiation fields without extra radiation or invasive imaging.

**Aim:** Our objective was to provide a biological experimental basis for monitoring matching of adjacent radiation fields between photon and electron fields due to introduced shifts during radiotherapy by CLI technique.

**Approach**: A medical accelerator was used to generate photon and electron fields. An industrial camera system was adopted to image the excited CLI signal during irradiation of chicken tissue with yellow (group A and group C experiments) or black color (group B experiment). The following introduced shifts were tested: 10, 5, 2, and 0 mm toward superior or inferior direction. A model was introduced to deal with matching error analysis of adjacent radiation fields due to introduced shifts with adapted plans used to treat neoplasms of the right breast with supraclavicular nodes or internal mammary lymph node.

**Results:** The matching values between photon and electron fields were consistent with the tested introduced shifts during yellow chicken irradiation. In group A, average discrepancies were 0.59±0.35  mm and 0.68±0.37  mm for photon fields and electron fields in anterior/posterior (AP) direction, with 87% and 75% of measurement within 1 mm, respectively. In group C, average discrepancies were 0.80±0.65  mm and 1.07±0.57  mm for oblique photon field with gantry angles of 330 deg and 150 deg, with 66% and 65% of measurement within 1 mm, respectively. The average discrepancies were 0.44±0.30  mm for electron field in the AP direction, with 94% of measurement within 1 mm. The matching error introduced by the proposed method was less than 1.5 mm for AP fields and 2 mm for oblique incidence fields. However, the field matching could not be monitored with black chicken tissue irradiation due to a weak CLI signal that could hardly be extracted from background noise in group B.

**Conclusions:** CLI is demonstrated for the quantitative monitoring of the field match line on light biological tissue phantoms and has potential for monitoring of field matching in surface tissue during breast cancer radiotherapy.

## 1 Introduction

Recently, the incidence of breast cancer is increasing year by year. Radiotherapy can significantly reduce the postoperative recurrence rate of breast cancer.^1^ The radiation treatment region for advanced breast cancer includes breast or chest wall combined with regional lymph nodes. Irradiation of internal mammary lymph node or supraclavicular fossa (SCF) lymph nodes is applied to improve local control and to reduce the incidence of symptomatic disease in this region.^2^ In a radiotherapy plan, photon fields irradiated for the breast and internal mammary lymph node significantly increased the irradiated volume of the affected lung, even with volume-modulated arc therapy technique, which could reduce the dose to critical structures compared to traditional techniques while maintaining conformal and homogenous doses with a target volume within a few minutes of treatment. Therefore, photon fields and single electron field have often been irradiated for breast and internal mammary lymph nodes, respectively,^3^^,^^4^ which could significantly decrease the irradiated volume of affected lung during advance breast cancer radiotherapy in our department. These adjacent fields need matching under complex geometrical regions. However, due to patients’ respiratory movements or involuntary body movements and irregular neck and chest contours, in addition to soft and shifty breast tissue during breast cancer radiotherapy, the mismatched adjacent fields in surface exposure regions could result in insufficient dosage or overdose in these regions, which may lead to tissue injury, excessive skin burns, and potential death.^5^^,^^6^

Nowadays, image-guided radiation therapy (IGRT) technique is developing rapidly, aiming to decrease the deviation of irradiation position caused by tumor region deformation or movement during radiotherapy.^7^^,^^8^ It combines a medical accelerator and modern imaging equipment and monitors tumor displacement through collecting patients’ image information before or during treatment [such as cone beam computer tomography (CBCT),^9^^,^^10^ electronic portal imaging device (EPID),^11^^,^^12^ surface scanning,^13^^–^^15^ tumor marker of electromagnetic signal,^16^^–^^18^ and ultrasound-guided scanning^19^^–^^21^] in order to precisely irradiate the tumor region. However, the projection of rays into the patient’s surface cannot be monitored by the above techniques. Moreover, the ionization chamber, film, and diode have been available for measuring projection of rays into the patient surface, but certain problems still exist in measured materials, such as no good tissue equivalence, low spatial resolution, invasive measurement, and no field matching measurement. The matching issue of adjacent radiation fields is still unsolved. It has become a hot and difficult issue about how to achieve monitoring matching of adjacent radiation fields during radiotherapy and reduce various side effects caused by mismatching.

Cherenkov emission is emitted when a charged particle traverses a dielectric medium with a velocity greater than the phase velocity of light in the medium.^22^ Cherenkov luminescence imaging (CLI) is an optical imaging modality to study charged particles of sufficient energy exceeding the Cherenkov emission light produced in biological tissue.^23^^–^^26^ With a typical continuous spectrum in biological tissue, the emitted light is then highly scattered and absorbed before reaching the surface, and the tissue optical properties tend to favor the transmission of the red-infrared light, where the Cherenkov emission is minimal. Due to the low light level, the detection of Cherenkov luminescence typical requires a charge-coupled device (CCD) coupled to a focusing optics lens, which is placed in a light-tight environment. Compared with traditional IGRT technique, CLI has many advantages, such as no radiation damage, fast imaging speed, high throughput, high spatial resolution, high sensitivity, low cost, and a wide range of applications, especially in monitoring the location of radiation rays in the body surface during radiotherapy. It can be used to effectively detect the matching information of adjacent radiation fields without extra radiation or invasive imaging. Therefore, CLI has a good application prospect in field matching in surface exposure regions during biological tissue radiotherapy.

In the context of radiotherapy, CLI has the unique capability of observing changes in the radiation fluence during treatment delivery and detecting the matching information of adjacent radiation fields.^27^^,^^28^ However, there are some unsolved issues for monitoring matching information of adjacent radiation fields with the CLI method. First, previous studies on field matching mainly discussed the accuracy of beam delivery without considering field mismatching projected in a surface exposure region due to introduced shifts. Second, previous works have been originally demonstrated in solid phantoms without considering the influence of biological tissue with an irregular surface and different optical properties.^29^^–^^32^ Third, matching has only been reported between photon and photons fields and electrons and electron fields without considering matching between photon and electron fields,^33^ which often occurs in advanced breast cancer radiotherapy. Finally, previous works have not considered the effect of high dose rate photon fields’ projection on the field matching. Therefore, the application of CLI in monitoring the matching of radiation fields during radiotherapy still needs to be studied. In this work, in view of the above unsolved issues, the matching of adjacent radiation fields was monitored quantitatively by CLI during irradiation of chicken tissue with yellow or black color. A model was introduced to quantify the matching error. The aim of this paper is to provide a biological experimental basis for monitoring matching of adjacent radiation fields due to introduced shifts during radiotherapy by the CLI technique.

## 2 Methods

### 2.1 Radiation Delivery

To explore the monitoring ability of CLI for the matching between photon and electron fields, all radiation treatment fields were delivered using a medical accelerator (Elekta VersaHD, Stockholm, Sweden) with CBCT. Cherenkov emission photons were captured using a camera with CCD during irradiation of chicken surfaces with yellow or black color. The generated photon energies included 6 and 10 MV of conventional a dose rate of 600  MU/min and 6 and10 flattening filter free (FFF) of high dose rate. The dose rate of 6 and 10 FFF were 1200 and 2400  MU/min, respectively. The generated electron energies included 4, 6, 8, 10, 12, and 15 MeV with a dose rate of 600  MU/min. The experiments were divided into two parts and three groups.

**Fig. 1:**
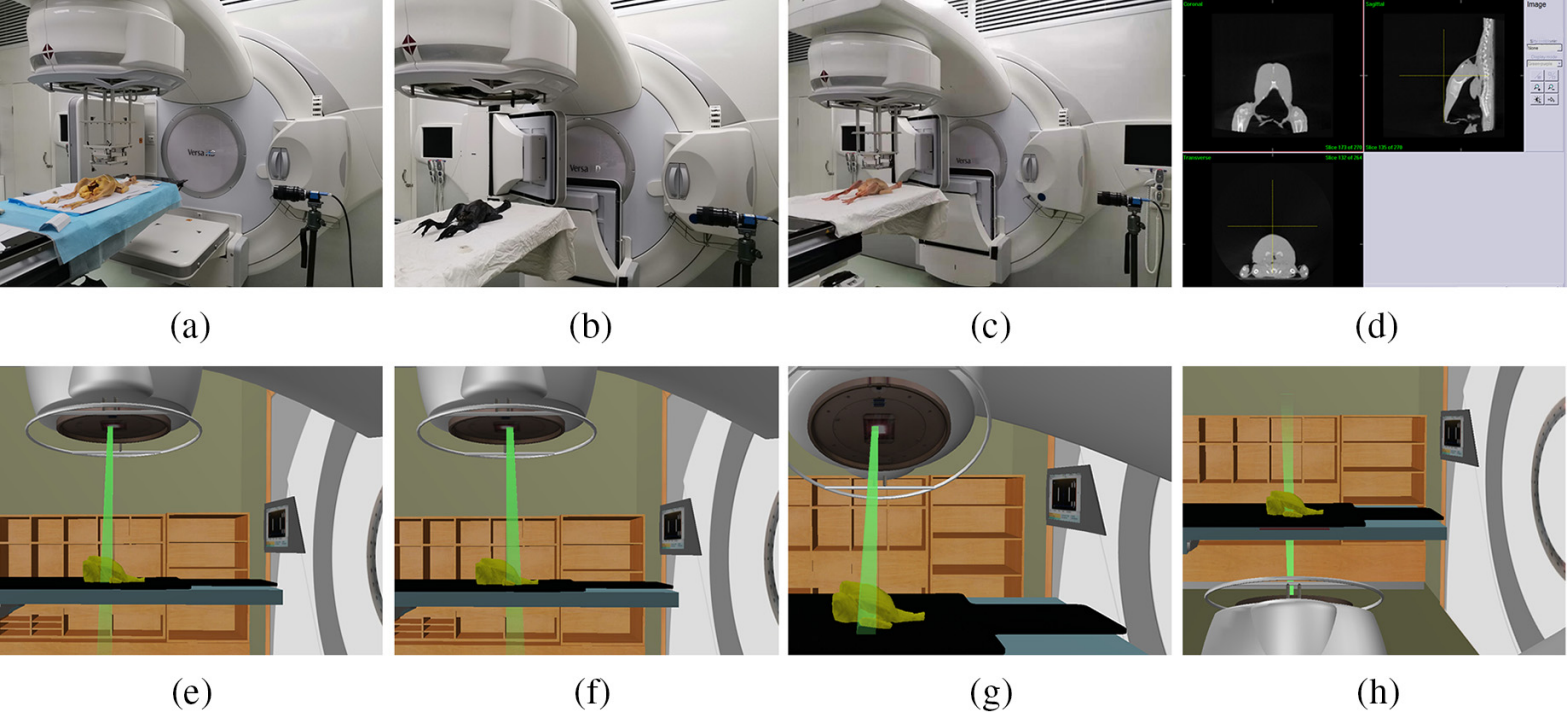
The set up for monitoring matching between photon and electron fields. (a) Group A: yellow chicken experiment. (b) Group B: black chicken experiment. (c) Group C: yellow chicken experiment according to conventional breast radiotherapy plan. (d) CBCT image with three-direction plane. (e) Schematic illustration for photon field. (f) Schematic illustration for electron field. (g) Schematic illustration for photon field with gantry angle of 330 deg. (h) Schematic illustration for photon field with gantry angle of 150 deg.

Part 1: the matching between photon and electron fields in the anterior/posterior (AP) direction in the surface of irradiated yellow chicken (group A) and black chicken (group B). In these experiments, the chicken was symmetrically placed on the accelerator treatment bed in supine position. The irradiation fields were divided into a lower photon irradiation field [Fig. 1(e)] and an upper electron irradiation field [Fig. 1(f)]. The photon field was delivered using collimator jaws set to $5\times 5\;{\mathrm{cm}}^{2}$ at isocenter and 101.7 cm source-to-surface distance (SSD) [Fig. 1(e)]. the electron field was delivered using a $10\times 10\;{\mathrm{cm}}^{2}$ cone with a $5\times 5\;{\mathrm{cm}}^{2}$ field. The SSD for the electron field was set the same as the photon field to ensure that the machine head will not touch the chicken, as shown in Figs. 1(e) and 1(f). Photon–electron matched fields were delivered with known introduced shifts established using calculated table shifts. The following introduced shifts were tested: 10, 5, 2, and 0 mm toward inferior or superior direction using a couch motion.

Part 2: the matching between photon and electron fields in the surface of irradiated yellow chicken according to conventional breast radiotherapy plan (group C). In these experiments, the yellow chicken was symmetrically placed on the accelerator treatment bed in a supine position. The on-board imager integrated in the medical accelerator was adopted to acquire CBCT images before irradiation. The scan parameters for generating middle resolution images were as follows: 120 kV, 300 mAs, S10, and F0. The scan angle was from −178  deg to 178 deg. The CBCT images were reconstructed with a thickness of 3 mm [Fig. 1(d)] and then transmitted to a Monaco V5.1 treatment planning system (Elekta, Stockholm, Sweden). To simulate the breast or chest wall irradiation and SCF or internal mammary lymph node irradiation for a breast cancer radiotherapy plan, the tangent photon fields of angles 330 deg and 150 deg were delivered to the chicken chest wall region, and a single electron field with an AP direction was delivered to the chicken neck region [Figs. 1(f)–1(h)]. The fields’ isocenters were the same as the CBCT scanning center in order to ensure that an isocenter was adopted for all fields and the machine head would not touch the chicken [Figs. 1(g) and 1(h)]. All photon fields were delivered using $5\times 5\;{\mathrm{cm}}^{2}$ collimator jaws at the isocenter and all electron fields were delivered with $5\times 5\;{\mathrm{cm}}^{2}$ field using a $10\times 10\;{\mathrm{cm}}^{2}$ cone. After the plan was completely designed, it was transmitted to the Mosaiq network system and executed on the accelerator. Photon–electron matched fields were delivered with known introduced shifts established using calculated table shifts. Given that typical field junctions of 5 mm are employed clinically,^34^ the ability to verify field matching within 2 mm is ideal. Therefore, the following introduced shifts were tested: 10, 5, 2, and 0 mm toward inferior or superior direction.

### 2.2 Image Acquisition

An industrial camera system (DMK 23U274) with a CCD was positioned to image the CLI signal in the chicken surface during irradiation. The camera was equipped with a fixed focal length and high aperture lens, which was fixed by a tripod [Fig. 1(a)]. In group A and group B, the surface of the lens was 80 cm away from the isocenter of the accelerator machine. In group C, the surface of the lens was 130.5 cm away from the isocenter of the accelerator machine in order to avoid colliding with the accelerator head during CBCT scanning. To decrease the noise signal and avoid CLI signal interference from surrounding lights, light-blocking tape was used to shield all light-emitting devices in the treatment room, which included fluorescent lamps, safety lights, air conditioning indicator lights, an EPID plate sign lamp, an x-ray volumetric imager detection plate lamp, the control box lamp of a bed hand, a display lamp, and ultrasound-guided head lights on the ceiling

Before the beginning of the image acquisition, the ruler with a standard scale was imaged at the accelerator isocenter plane and the corresponding relationship between the scale and the photo pixel was be established for quantitative analysis about the matching error. In the process of image acquisition, the highest gain of 36 dB and minimum framerate of 5  f/s were selected to obtain a high signal-to-noise ratio during image acquisition. To optimize the integration acquisition time, the integration time was increased until the maximum signal intensity was observed. This verified that a minimum integration time of 1 s for photon irradiation and 1.2 s for electron irradiation were optimal to capture a maximum amount of Cherenkov signal. However, CLI signals could not be detected when the above integration time was adopted in group B. Next, the integration time was increased until relatively clear CLI signal images could be obtained. This verified that a minimum integration time of 2 s for photon irradiation and 2.5 s for electron irradiation were optimal to capture relatively clear CLI signal images in group B.

### 2.3 Matching Error Analysis

**Fig. 2:**
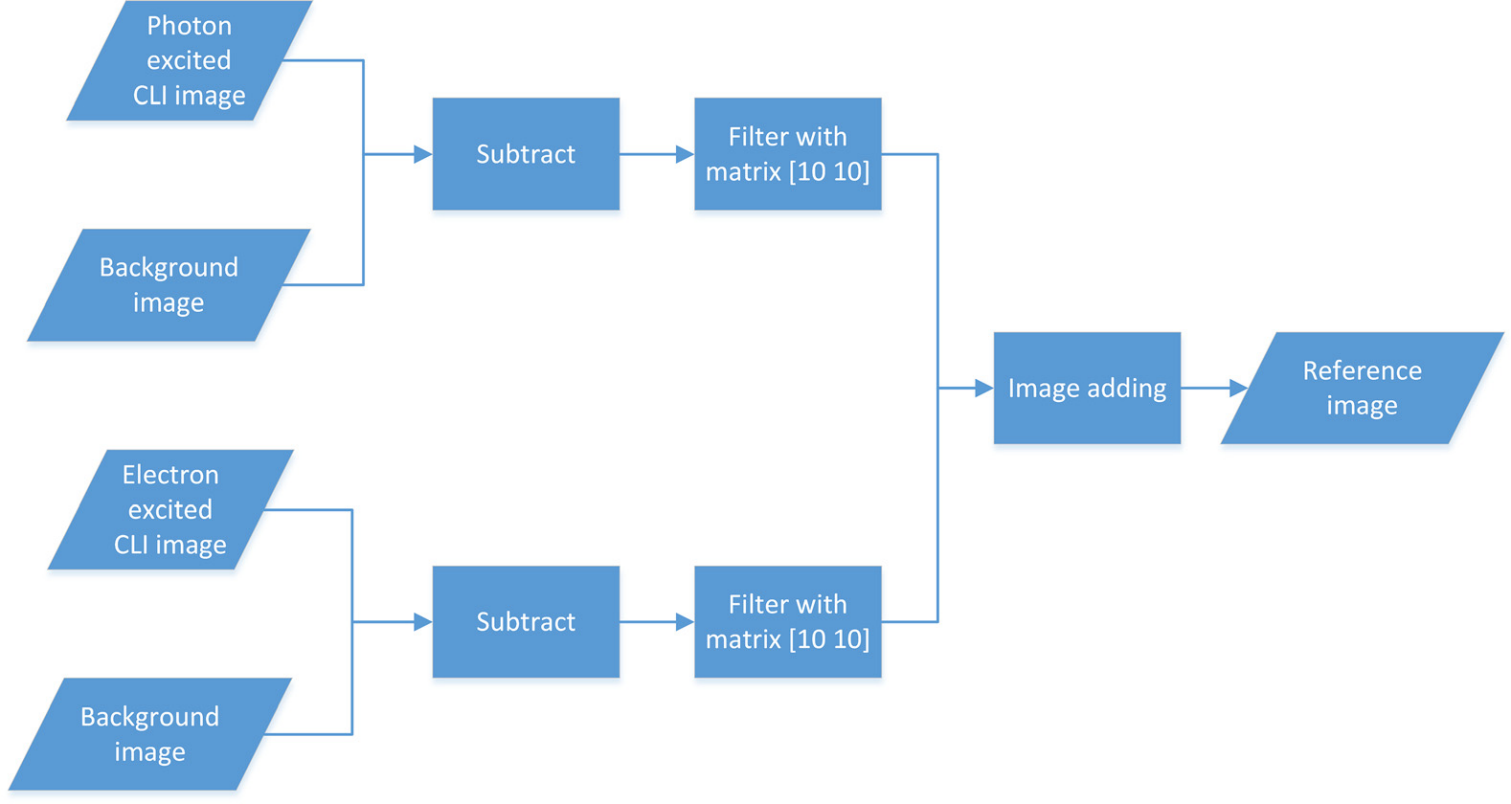
With no introduced shifts, the photon-excited CLI image was added to the electron-excited CLI image of no introduced shifts as the reference image.

**Fig. 3:**
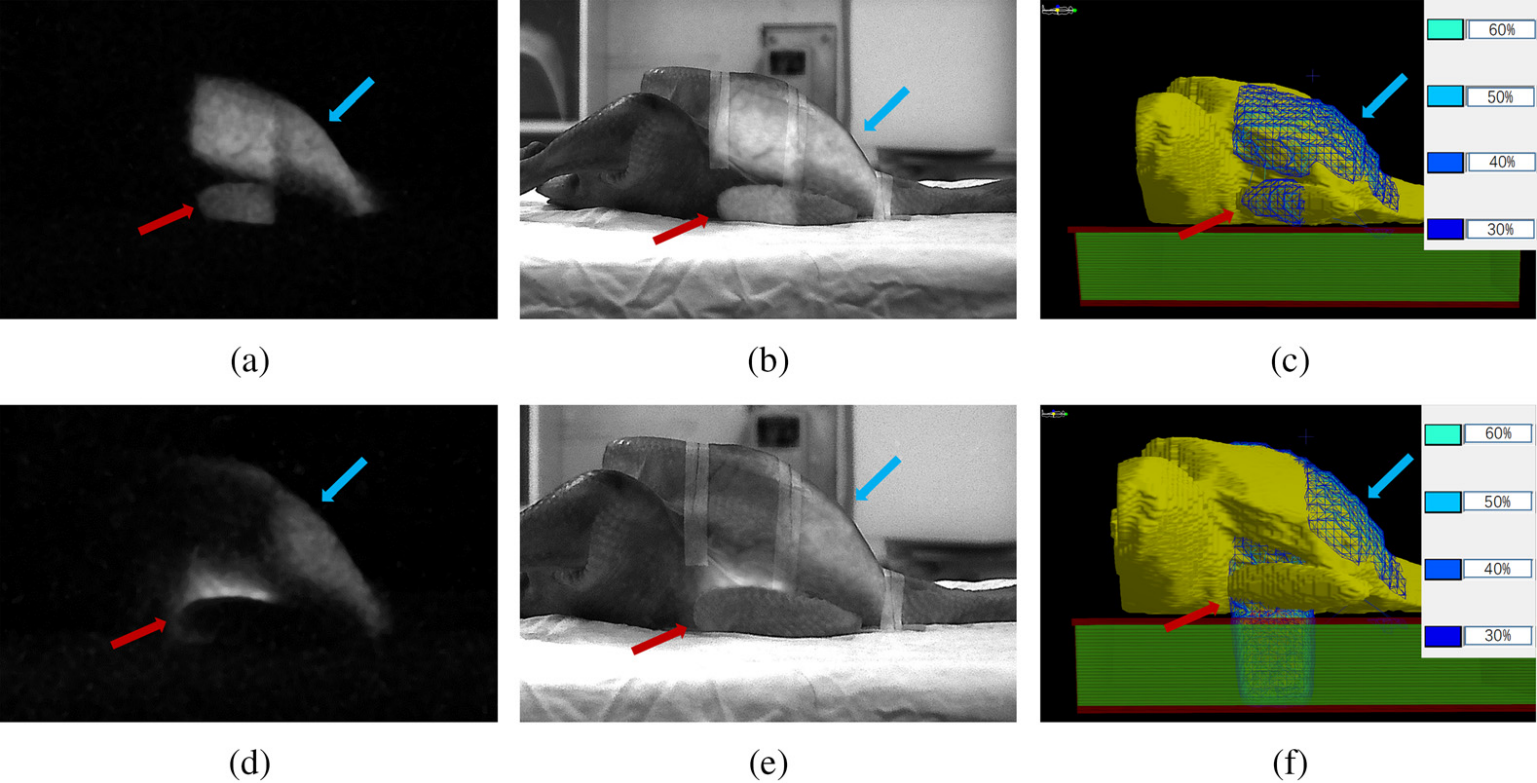
Added and fused images in Group C for photon fields with gantry angles of 330 deg and 150 deg. (a), (d) The reference CLI image between photon and electron fields (600 MU for photon field and 720 MU for electron field). (b), (e) The fused image of reference CLI image and photograph. (c), (f) Calculated dose distribution in the chicken surface from TPS. Red or blue arrows pointed toward the position and shape of chicken wing or chest wall silhouettes in the CLI image, fused image, and calculated dose image, respectively.

All images were processed and evaluated using MATLAB^®^ software packages (R2015b). The background images were acquired with the same conditions before the irradiation (radiation off) and subtracted out of the measured CLI image during the process of image analysis. To remove the sparkle noisy pixels caused by high-energy photons hitting the CCD directly, each image was generated by median filtering over a stack of three images from repetitive measurements and then smoothed by a median filter with a kernel size of 10  pixels×10  pixels.^35^ First, with no introduced shifts, the photon-excited CLI image was added to the electron-excited CLI image as a reference image, as shown in Fig. 2. Second, reference images were added to the photographic view taken at the same time [Figs. 3(b) and 3(e)]. According to the red and blue arrows as shown in Figs. 3(b) and 3(e), the location and shape of chicken wings and chest wall silhouettes in reference to Cherenkov image were consistent with that in photographic view images, which indicated the accuracy of the reference image (Fig. 3). By comparing the fused image and calculated dose image as shown in Figs. 3(b), 3(c), 3(e), and 3(f), CLI signals in the reference images showed similar locations and shapes with the chicken surface dose calculated by the treatment planning system (TPS) (Fig. 3). Finally, it could be seen that the location and shape of the CLI signal were consistent with that of the treatment region and surface dose.

**Fig. 4:**
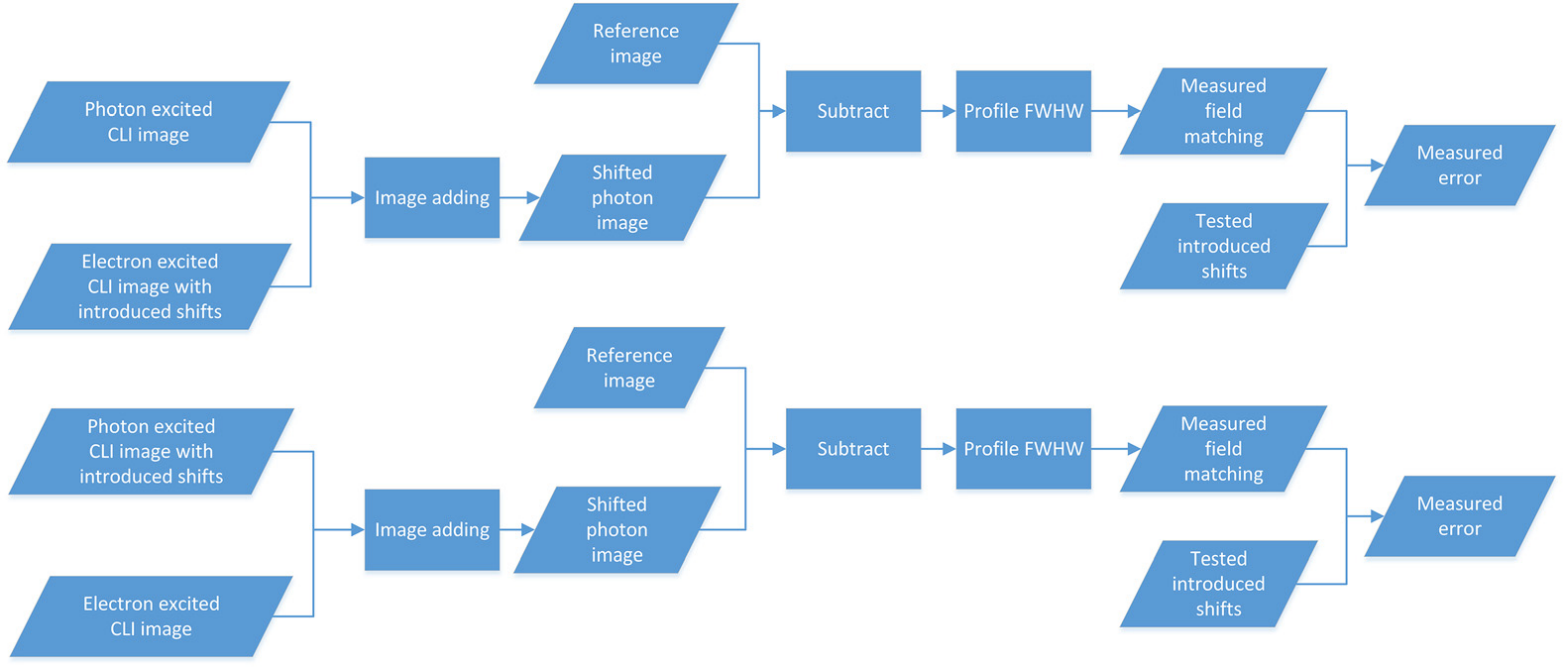
Matching error measurement for photon and electron fields.

**Fig. 5:**
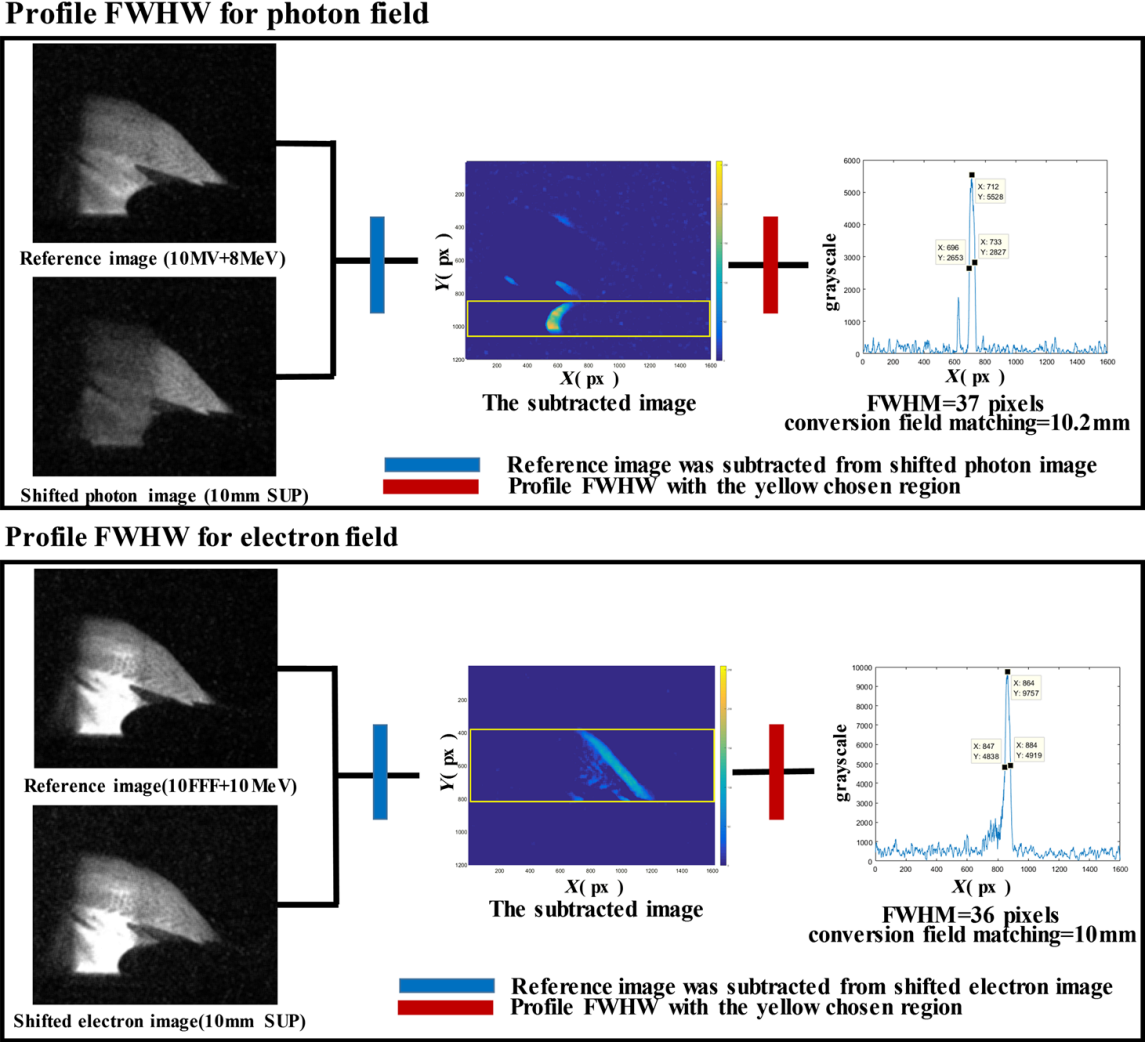
Calculated process of profile FWHM and corresponding measured shifts due to introduced shifts during photon or electron field delivery.

Fields were delivered with known introduced shifts. Changes in the Cherenkov intensity represented field-matching values between photon and electron fields, which was evaluated through the generation of difference mismatching images from reference images. The photon-excited CLI image with tested introduced shifts was added to the electron-excited CLI image of no introduced shifts as a shifted photon CLI image. The electron-excited CLI image with tested introduced shifts was added to the photon-excited CLI image of no introduced shifts as a shifted electron CLI image. Then the reference image was subtracted from the shifted photon or electron CLI image to obtain the field matching error (Fig. 4). As shown in Fig. 5, in the matching error measurement for the photon field, a solid yellow rectangle region was placed on the location of the chicken wing in the subtracted image. The profile and the corresponding full width at half maximum (FWHM) of the chosen region along the X axis direction could be calculated using MATLAB^®^ software and indirectly represented the matching result due to lower chest wall movement during photon field delivery. In the matching error measurement for the electron field, the solid yellow rectangle region was placed on the location of the chicken chest wall in the subtracted image. The profile FWHM of the chosen region profile along the X axis direction could be calculated and indirectly represented the matching result due to upper chest wall movement during electron field delivery. The FWHM was calculated manually as the pixel length width at half maximum intensity in grayscale profile. The maximum intensity and corresponding half maximum value were chosen manually using the MATLAB^®^ data cursor tool in grayscale profile. With pixel-to-millimeter conversion factor, the FWHM was converted into millimeters as measured by the matching value.^36^ It was an easy and reliable method for calibrating distances in a clinical situation without the need for an additional device. In the results provided below, all matching values are absolute discrepancies in mm.

## 3 Results and Discussion

### 3.1 Result for Mean Grayscale Value of CLI Images

**Fig. 6:**
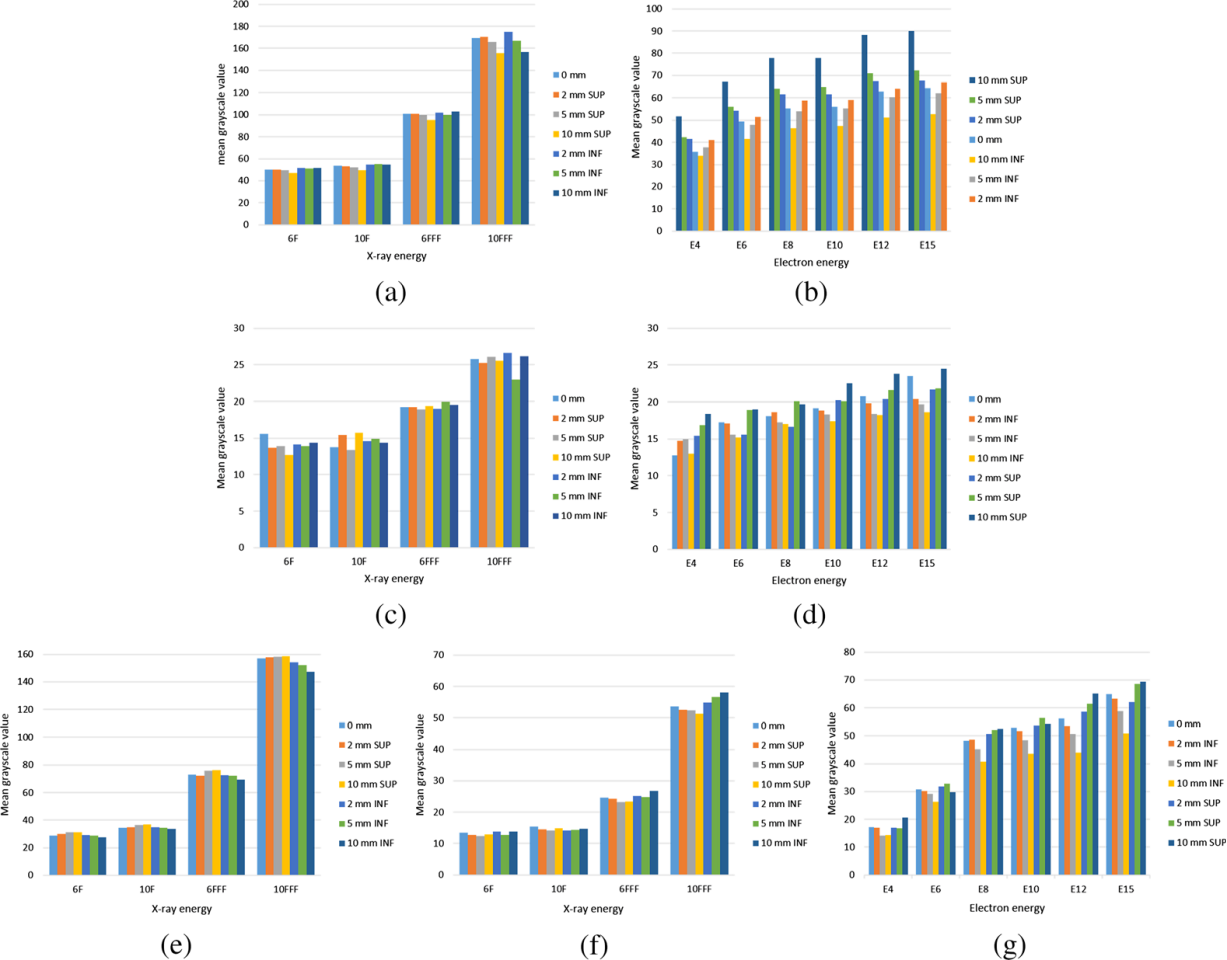
MGV of photon and electron fields in the surface of irradiated yellow and black chicken (INF movement indicated chicken moved toward inferior direction. SUP movement indicated chicken moved toward superior direction.) (a) Photon field results in group A, (b) electron field results in group A, (c) photon field results in group B, (d) electron field results in group B, (e) photon field results with a gantry angle of 330 deg in group C, (f) photon field results with a gantry angle of 150 deg in group C, (g) electron fields in group C.

**Fig. 7:**
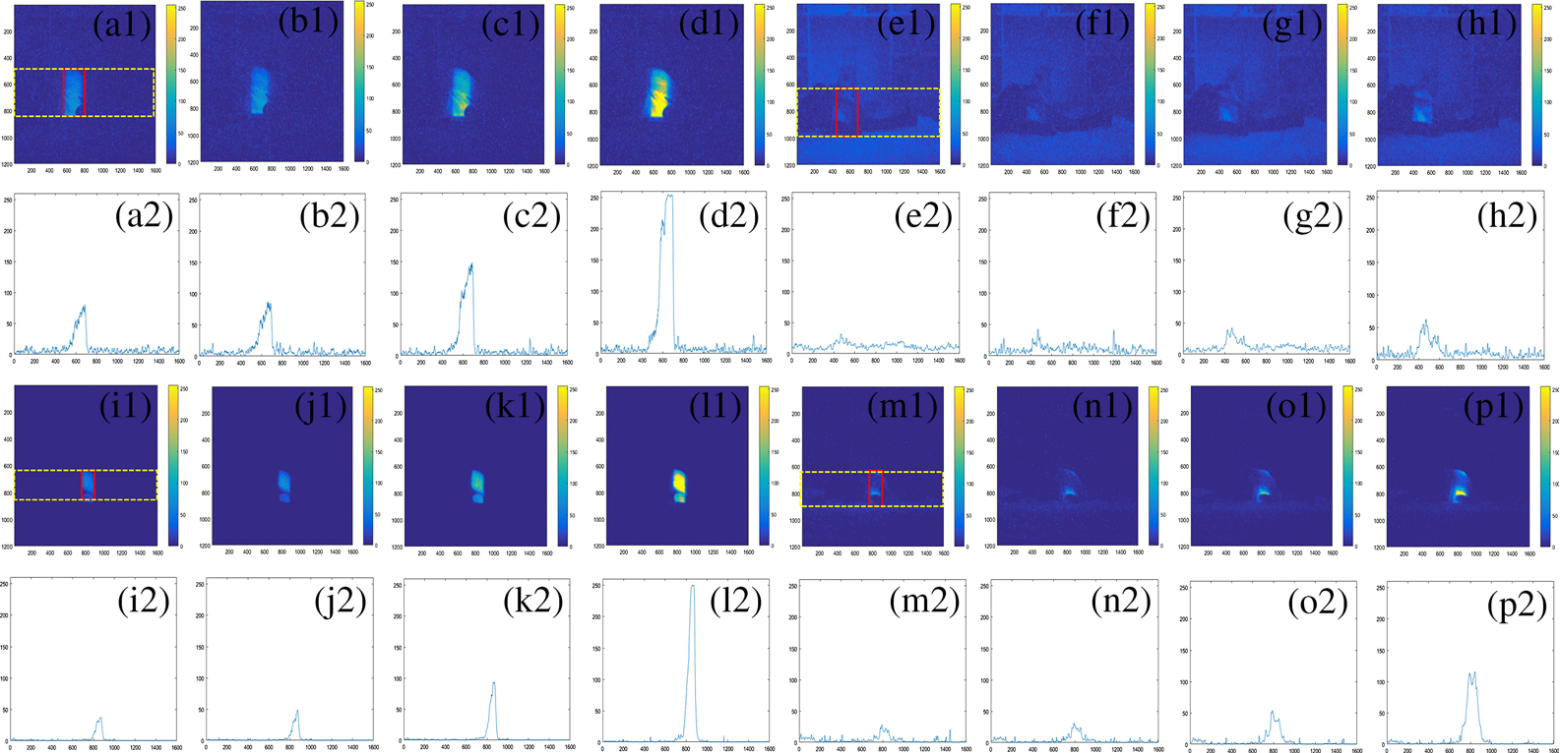
CLI images and corresponding grayscale profile with the yellow dotted region for photon-excited CLI images in group A [(a1), (a2) 6 MV; (b1), (b2) 10 MV, (c1), (c2) 6 FFF; and (d1), (d2) 10 FFF], group B [(e1), (e2) 6 MV; (f1), (f2) 10 MV; (g1), (g2) 6 FFF; and (h1), (h2) 10 FFF], group C with a gantry angle of 330 deg [(i1), (i2) 6 MV, (j1), (j2) 10 MV; (k1), (k2) 6 FFF; and (l1), (l2) 10 FFF], and group C with a gantry angle of 150 deg [(m1), (m2) 6 MV; (n1), (n2) 10 MV; (o1), (o2) 6 FFF; and (p1), (p2) 10 FFF].

Mean grayscale value (MGV) is defined as mean grayscale value of a chosen $5\times 10\;{\mathrm{cm}}^{2}$ (with pixel-to-millimeter conversion) rectangle region for the photon field and a chosen $5\times 7.5\;{\mathrm{cm}}^{2}$ (with pixel-to-millimeter conversion) rectangle region in the center of the CLI image. The chosen region was illustrated as a solid red rectangle region as shown in Figs. 7(a1), 7(e1), 7(i1), 7(m1), and 8(a1), 8(g1), 8(m1). The MGV was calculated by averaging all the pixel values belonging to the chosen region using MATLAB^®^ software. For electron fields, the MGV of images increased with the increase of energy (Fig. 6). According to the percent depth dose measured with parallel plate ionization chamber for the electron beam, the superficial dose is linear to the electron beam energies. The higher the energy, the higher the surface dose. Therefore, it was consistent with research by Zhang et al.,^30^^,^^37^ which concluded that the Cherenkov signal intensity was directly proportional to the reference superficial dose data of corresponding electron energies simulated by the Monte Carlo method. For conversional dose rate photon fields, there was no obvious MGV difference with the increase of photon energy. However, the MGV for high dose rate fields was larger than that for conversional dose rate fields. This could be due to the high dose rate for 6 and 10 FFF fields, which were 2.33 to 3.66 times higher than that for conversional dose rate fields, which deposited larger superficial doses and excited larger Cherenkov photons per minute, resulting in larger MGV of the CLI image for high dose rate photon fields. Therefore, in the case of obtaining the same CLI intensity, integration time for high dose rate fields could be greatly reduced compared to that for conversional dose rate fields. Field matching efficiency for high dose rate fields could be greatly increased. The MGV results provide a reference for the selection of energy and efficiency assessment in field matching during radiotherapy.

**Fig. 8:**
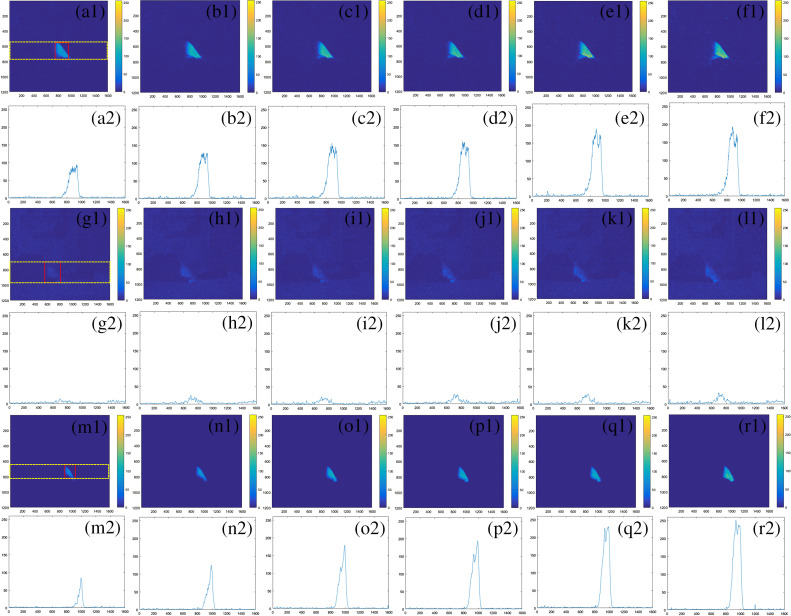
CLI images and corresponding grayscale profile with the yellow dotted region for electron-excited CLI images in group A [(a1), (a2) 4 MeV; (b1), (b2) 6 MeV; (c1), (c2) 8 MeV; (d1), (d2) 10 MeV; (e1), (e2) 12 MeV; and (f1), (f2) 15 MeV], group B [(g1), (g2) 4 MeV; (h1), (h2) 6 MeV; (i1), (i2) 8 MeV; (j1), (j2) 10 MeV; (k1), (k2) 12 MeV; and (l1), (l2) 15 MeV], and group C [(m1), (m2) 4 MeV; (n1), (n2) 6 MeV; (o1), (o2) 8 MeV; (p1), (p2) 10 MeV; (q1), (q2) 12 MeV; and (r1), (r2) 15 MeV].

In three experiments, the MGV of the CLI image for electron fields was greater than that for conversional dose rate photon fields, which was due to the deeper depth at max doses (roughly 1.5 cm for 6 MV and 2.0 cm for 10 MV) for conversional dose rate photon fields, resulting in higher counts for electron fields. The MGV for photon fields with a gantry angle of 150 deg was less than that for photon fields with a gantry angle of 330 deg. This could be caused by decreased Cherenkov photons collected from the exit side of the photon radiation. Different introduced shifts have great influence on the MGV for electron fields in the three experiments (Fig. 6). This was due to the variational contact areas including neck and upper chest wall between irradiation and the chicken surface, which were greatly affected by the different introduced shifts, resulting in a great variation in the number of Cherenkov photons under different introduced shifts for electron fields.

The grayscale profile value of CLI images (GPVCI) was defined as the averaged intensity profile value of a chosen rectangle region for photon fields and electron fields. The chosen region was illustrated as a dotted yellow rectangle region as shown in Figs. 7(a1), 7(e1), 7(i1), 7(m1) and 8(a1), 8(g1), 8(m1). The GPVCI for conventional dose rate photon fields was less than that for all electrons fields in group A (Fig. 7). This was consistent with the previous reports, which demonstrated that Cherenkov photons for photon fields were generated by secondary electrons through Compton scattering or photoelectric interaction and Cherenkov photons conversion efficiency for photon fields was lower than that for electron fields^38^ resulting in the above phenomenon. Compared with the results in group A, the MGV and GPVCI in group C were less for all photon and electron fields (Figs. 6–8). With the increase of CCD detection distance in group C, the attenuation distance increased in the air for excited Cherenkov photons and the Cherenkov photons reaching the CCD detector decreased, resulting in decreases of MGV and GPVCI in group C. In addition, the GPVCI outside the chosen rectangle region in group C was significantly less than that in group A for electron fields and photon fields with a gantry angle of 330 deg which indicated that the noise may be attenuated greatly as the detected distance increased (Figs. 7 and 8).

### 3.2 Result for Field Matching

**Fig. 9:**
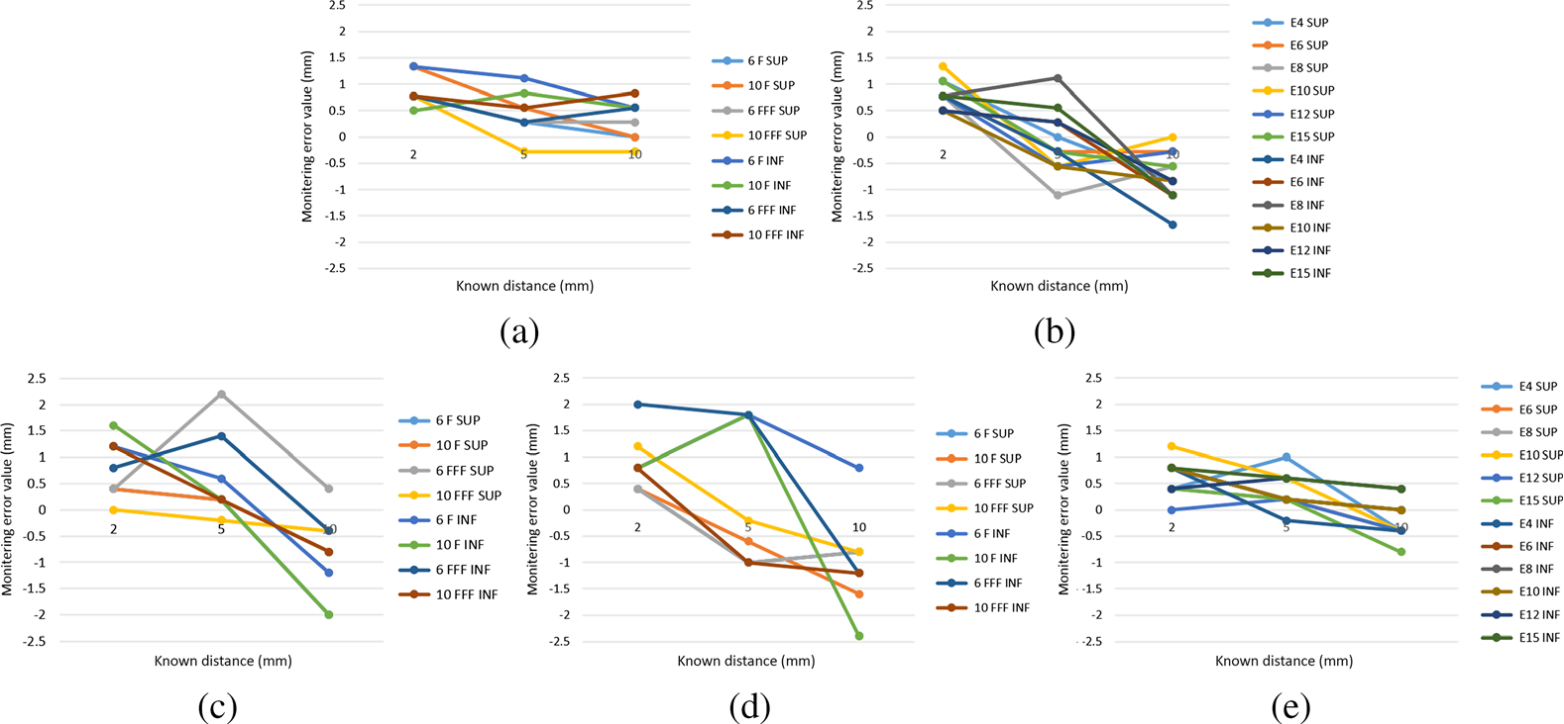
Matching error values for photon fields and electron fields in surface of irradiated yellow chicken (INF movement indicated chicken moved toward inferior direction. SUP movement indicated chicken moved toward superior direction.) (a) Photon fields results in group A, (b) electron field results in group A, (c) photon field results with a gantry angle of 330 deg in group C, (d) photon field results with a gantry angle of 150 deg in group C, and (e) electron field results in group C.

In group A, the measured matching values were consistent with the tested introduced shifts values and all matching errors were within 1.5 mm (Fig. 9). Average discrepancies were 0.59±0.35  mm for photon fields with 87% of measurements within 1 mm. Average discrepancies were 0.68±0.37  mm for the electron field with 75% of measurements within 1 mm. These errors appeared to have no energy dependence in group A. In group C, the measured matching values were consistent with the tested introduced shifts (Fig. 9). In the majority of experiments of group C, the matching error values were within 2 mm for photon fields and 1.5 mm for electron fields. However, the matching error value was 2.2 mm for 6 FFF fields with 5 mm toward superior movement in the oblique incident field with a gantry angle of 330 deg. The matching error was 2.4 mm for the 10-MV field with 10 mm toward inferior movement in the oblique incident field with a gantry angle of 150 deg. The average discrepancies were 0.80±0.65  mm and 1.07±0.57  mm for photon fields with gantry angles of 330 deg and 150 deg with 66% and 65% of measurements within 1 mm, respectively. The average discrepancies were 0.44±0.30  mm for electron fields with 94% of measurements within 1 mm. These matching errors were not energy dependent in group C.

Under AP irradiation in group A, the matching error values were within 1.5 mm for all photon fields and electron fields. Under oblique angles’ irradiation in group C, all matching error values were within 2 mm for photon fields, with all but two being within 1.5 mm for electron fields. For electron fields, the matching error values in group C were more than that in group A (Fig. 9). The matching error values for AP photon fields were less than that for electron fields in group A. However, in group C, the matching error values for oblique photon fields were more than that for electron fields. These results meant that the agreement between tested introduced shifts and measured values could be reduced with increasing field obliquity for photon fields. Earlier work by Zhang et al.^39^ has similar results, which demonstrated that this was due to the oblique field delivery, which extended the high-intensity field area in the direction of the field and broadened the field edge, effectively blurring the Cherenkov field edge and resulting in increased matching error values. These discrepancies, however, appeared to not be energy dependent. Interestingly, although the matching error values for electron fields were all within 1.5 mm in group A and group C, the proportion less than 1 mm was significantly higher in group C with the same irradiation conditions. This could be caused by a decrease of the noise signal in the CLI image when the CCD detection distance increased in group C, which made the field boundary signal clearer and improved the detection accuracy. Additionally, it appeared to have downward trends with respect to larger introduced shifts in Fig. 9. The CLI intensity edge would be broadened due to signal noise and blurring CLI image, which mostly resulted in more matching value than introduced shifts in this study. It was most obvious for small introduced shifts. The matching discrepancies were consistently positive values for 2-mm introduced shifts in group A and group C. The impact of a broadening edge has been attenuated when introduced shifts decreased due to greater calculated cardinality, which resulted in downward trends with respect to larger introduced shifts.

It is important to note that for the AP irradiation photon field in group A, discrepancies were consistently positive values with average value of 0.55 mm. This systematic discrepancy was observed for all introduced shifts and energies in group A, which indicated that a correction factor could be determined to decrease matching error values. The matching error could be less than 1 mm for 2-mm introduced shifts if the calculated error is subtracted from the systematic discrepancy for the AP photon field in group A. However, it was a limitation that a more percent error appeared for 2-mm introduced shifts by the proposed method. It may be caused by low image resolution (36  pixels=10  mm) with the CCD, which resulted in a larger pixel value of the profile FWHM and ultimately a larger percent error. Future works would focus on the high-resolution camera for small introduced shifts and we are striving for a much smaller percent error. However, in the results of all oblique irradiation photon fields in group C, there was no consistent discrepancy. This could be caused by the oblique field delivery, which extended the high-intensity field area in the direction of the field and broadened the field edge, resulting in a different deviation direction for matching error values.

### 3.3 Result for High Dose Rate Photon Field

In three experiments, the MGV and GPVCI for high dose rate fields were larger than that for conversional dose rate fields (Fig. 6). This could be due to the high dose rate for 6 and 10 FFF fields at 2.33 to 3.66 times higher than that for conversional dose rate fields, which deposited larger superficial doses and excited larger Cherenkov photons, resulting in larger MGV of the CLI image for high dose rate photon fields. The higher the dose rate, the greater the MPV and GPVCI for high dose rate fields. In oblique irradiation photon fields, it was found that the variance and deviation distribution range of matching error values were less for high dose rate photon fields than that for conventional dose rate fields. These results could be explained by a larger deposited superficial dose and larger excited Cherenkov photons, which effectively increased the Cherenkov intensity and make the field edge clearer, resulting in matching values close to the tested introduced shifts for high dose rate photon fields.

Compared with the dose profile of conversional dose rate energies (6 and 10 F) in water, a higher dose region appeared in the middle profile of the high dose rate energies (6 and 10 FFF). Details about the dose profile information are illustrated in Fig. S1 in the Supplementary Material. However, this did not happen in the GPVCI for photon fields, with no obvious distribution difference between high dose rate photon fields and conventional dose rate photon fields (Fig. 7). This could be due to small fields and irregular biological surface structures, which could weaken the signal of the high dose region for high dose rate photon fields and ultimately result in the above phenomenon.

### 3.4 Effect of Color on Matching Result

The Cherenkov effect produces a broad spectrum of light emission from UV down to near-infrared. In tissue, the emitted light is highly scattered and absorbed before leaving the surface and ultimately is collected by the CCD with a sensitive range in the visible wave band in this study. When the color of chicken tissue was black in group B, Cherenkov photons were highly absorbed by dark tissue with a high absorbed coefficient of the visible band, which resulted in dominance of Cherenkov emission in the spectrum of black tissue out of the sensitivity range of the CCD camera used and ultimately Cherenkov photons were hardly collected by the CCD with the same integration time as the other two groups. However, when the integration time was doubled, the number of unabsorbed photons increased and a CLI intensity image could be obtained with low intensity, large image noise, and fuzzy CLI intensity boundary. Previous research by Andreozzi et al.^40^ has similar results, which demonstrated that the darker tissue absorbed most Cherenkov photons, resulting in a Cherenkov signal being hardly collected by CCD. With the integration time increased in group B, the noise signal increased in CLI images, especially for photon fields (Figs. 7 and 8), which was similar to the one observed by Zhang et al.^30^ and Andreozzi et al.^40^ As a result, due to the large image noise and fuzzy CLI signal boundary in group B, the CLI signal could not be extracted from the surrounding environment. The matching between photon and electron fields could not be monitored with black biological tissue irradiation.

## 4 Conclusions

We have shown that CLI was acquired for matching monitoring between photon and electron fields due to introduced shifts in a biological tissue during radiotherapy. This capability is enabled by imaging the excited Cherenkov intensity in chicken surface with yellow or black color using a CCD. We concluded that the matching values between photon and electron fields were consistent with the tested introduced shifts for photon and electron fields. However, it is a limitation that the mismatching errors were introduced only by shifting the chicken tissue due to introduced shifts during radiotherapy. Further work will be required for customizing the plans for mismatching error research. For advanced breast cancer patients, photon fields and a single electron field have often been irradiated for breast and internal mammary lymph nodes, respectively.^3^^,^^4^^,^^34^ These adjacent fields need matching under complex geometrical regions. However, due to patients’ respiratory movements or involuntary body movements during breast cancer radiotherapy, the mismatching between photon and electron fields could result in insufficient dosage or overdose in these regions. Therefore, the proposed technique may solve this problem and has the potential to apply to breast radiotherapy verification. Additionally, the proposed technique could be applied to craniospinal irradiation verification, which involves the matching of two whole brain fields and one to two spinal fields, depending on the anatomy of the patient. We believe, in the future, Cherenkov imaging could be incorporated into the treatment verification tools to monitor fields’ matching during every fraction radiotherapy and further improve on treatment delivery and accuracy in clinical situations.

## Code, Data, and Materials Availability

This study does not have associated code. Datasets generated during the present study were supported its results. All authors have declared the availability of data used in the research results reported in the paper and have provided specific access information or restrictions. No materials were used for the analysis in this study.
