# Supplementary material for: Using Cherenkov imaging to monitor the match line between photon and electron radiation therapy fields on biological tissue phantoms
Source: J Biomed Opt. 2020 Dec 9;25(12):125001. doi: 10.1117/1.JBO.25.12.125001 (PMC7725107; doi:10.1117/1.JBO.25.12.125001)
Supplement: Supplementary file 1 [file JBO_025_125001_SD001.pdf]

Supplemental Material

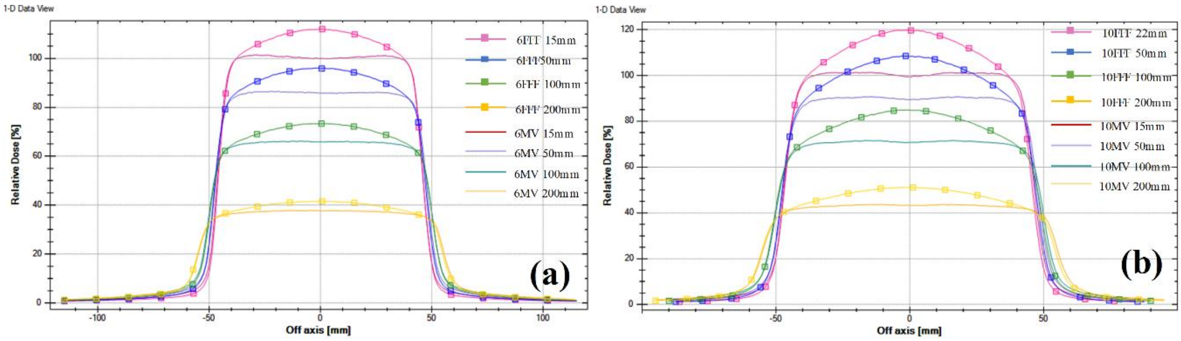

Fig. S1 The comparison between axial dose profile of high dose rate energies and axial dose profile of conversional dose rate energies measured with semiconductor in water tank under different depths (IBA Blue phantom2).

It is important to note that intensified CCD (ICCD) or electron multiplying intensified CCD (EM-ICCD) was adopted frequently to collect Cherenkov photon signal during radiotherapy in previous studies. However, the price of these devices was expensive. Most research groups maybe cannot afford. The CCD is an ordinary device and can be used for field matching research by any research group.

Compared with CCD, other types of camera can also image the excited CLI signals in chicken surface during radiotherapy and can be used for matching monitoring of adjacent fields, such as complementary metal oxide semiconductor (CMOS), ICCD, EM-ICCD camera. The decision of camera type depends on three main criteria to that application: (i) low light sensitivity, (ii) background light suppression, and (iii) fast frame rate. CCD and CMOS have similar property and maybe have similar results about matching monitoring. The linear accelerator used in this study delivers a 5 μs radiation pulse every 5ms. So the intensifier is able to act as a fast optical switch that activates when Cherenkov emission is expected during the pulsed radiation, which could increase the Cerenkov signal under the ambient room light by at least 1–2 orders of magnitude. ICCD or EM-ICCD with intensifier has ability to image the excited CLI signals with room lights on. Compared with CCD and CMOS, ICCD or EM-ICCD has high frame rate and high sensitivity, which allowed ICCD or EM-ICCD to collect the excited CLI signals from several or single radiation pulse. So ICCD or EM-ICCD can be used to detect field matching errors in real time. However, CCD or CMOS have high resolution (1600×1200 or 5184×3456) and small physical pixel size (4.4×4.4 μm or 4.3×4.3 μm). the lower resolution (512×512 or 1024×1024) and larger physical pixel size (16.0×16.0 μm or 12.8 × 12.8 μm) of the EM-ICCD or ICCD make intensity-mapped images smoother, which may increase matching error of adjacent fields compared with CCD or CMOS. CMOS and EM-ICCD have been not tested for verification of fields matching during radiotherapy in previous researches and could be investigated in future work.

Nowadays, dose distribution could be obtained from treatment plan system in the clinic. However, CLI distribution could not be obtained from the treatment plan system in the clinic. When the treatment plan was served as reference image, dose distribution image would be fused with CLI image during monitoring the match line between radiation fields. The fused error would be great as these images belong to different sources, which could result in great matching error. In order to eliminate the errors caused by different sources, it is necessary to convert the dose distribution to CLI distribution and then perform image fusion. According to recent published paper, dose distribution could be converted to CLI distribution using Monte Carlo simulated method (GEANT4 based GAMOS software). However, there is still an error in conversion process although the error is small. I believe the conversion error would be within clinical acceptance and the treatment plan could be used as reference image through dose-to-CLI

conversion in the future.
